# Meetings of Societies

**Published:** 1929

**Authors:** 


### Meetings of Societies.

### Bristol Medico-Chirurgical Society.

The first meeting of the session was held at the University on Wednesday, 10th October, 1928, when Dr. W. K. Wills resigned the chair to the new President, Dr. H. L. Ormerod.

DR. ORMEROD read his inaugural address on "**Mental Deficiency,**" reported on page 1 of this number.

The Report presented by the Honorary Secretary, MR. W. A. JACKMAN, showed that during the year 1927–28 eighteen new members had been elected and six resigned leaving a total membership of 209.

Eight meetings had been held during the session, four in the Society's room and four in the Physiological Lecture Theatre of the University. The average attendance had been fifty-five, an increase on that of the previous session, when the average attendance was forty-eight.

---

The second meeting of the session was held on Wednesday, 14th November, 1928, the President, DR. H. L. ORMEROD in the Chair.

MR. E. WATSON-WILLIAMS showed again the cases of carcinoma of the tonsil that he had brought before the Society in the previous session, to demonstrate the results of various methods of treatment.

MR. T. CARWARDINE opened a discussion on the "**Treatment of Duodenal Ulcer.**"

DR. R. C. CLARKE continued the subject from the medical aspect. These two papers were published on pp. 279, 289 of the *Journal*, Winter, 1928.

In the ensuing discussion MR. A. RENDLE SHORT remarked that the symptoms of duodenal ulcer were often anomalous, especially in women. The irregular meals of the latter perhaps accounted for some of this. In women, too, the differential diagnosis from gall-bladder disease might be very difficult. He thought that *pain* indicated an ulcer, while *discomfort* might mean only a reflex dyspepsia. Hemorrhage was by no means a constant feature of ulcer. Many patients turned to surgery because prolonged medical treatment failed to cure. The mortality of gastro-jejunostomy was 2 or 3 per cent. ; 85 to 90 per cent. of operations resulted in cure ; the failures were apt to appear at special clinics and produce a distorted view in those conducting the latter.

DR. CARLETON said that he found a mixture of chalk and magnesia in large doses relieved many cases of dyspepsia. The remedy, in addition to being efficacious, was cheap.

DR. VINCENT COATES advised the intensive alkali method of treatment for duodenal ulcer ; in his hands it had given 350 successive satisfactory results.

DR. L. N. MORRIS pointed out that the Sippey treatment gave excellent results, and that the patients could remain at work while it was carried on.

DR. D. A. ALEXANDER asked whether belladonna was less helpful than morphia after operation. He thought belladonna might be a factor in producing a post-operative parotitis, owing to the dryness of the mouth.

MR. A. W. ADAMS referred to the difficulty of making an early diagnosis and the possibility of malignant changes. If healing of an ulcer produced stenosis, a gastro-jejunostomy would be needed ; but the operation as a rule acted by alkalizing the stomach, and if it failed to do this the result was poor.

DR. O. BODMAN thought that too much attention was given to acidity, and not enough to the condition of the mucous membrane, which was perhaps often primarily at fault.

MR. C. A. MOORE thought the influence of anxiety and worry was considerable, and that the medical profession showed a high proportion of duodenal ulcers. He thought duodenal ulcer was frequent, and could generally be diagnosed on clinical evidence, without skiagraphy.

DR. J. R. CHARLES said that he found hydroxide of magnesia preferable to the carbonate. An objection to gastrectomy was the risk of achlorhydria, followed by pernicious anæmia.

———

The third meeting of the session was held on Wednesday, 12th December, 1928, the President in the Chair.

A demonstration was given of the use of the kinematograph in medical teaching. illustrated by films of surgical and pathological subjects.

———

The fourth meeting of the session was held on Wednesday, 9th January, 1929, the President in the Chair.

DR. BUSH showed a series of skiagrams illustrating pulmonary disease.

MR. A. W. ADAMS read a paper on "**The Rôle of Pyelography in Renal Disorders,**" reported on page 49.

DISCUSSION ON MR. ADAMS'S PAPER.

DR. BUSH spoke of the importance of proper preparation of the patient. The presence of gas in the intestines might be due to a badly-given enema. He agreed as to the value of pyelography, but for mere localization of stones other methods were possible ; for example, two pictures on one plate, one at inspiration, another at expiration. A lateral view similarly would assist. To skiagraph the kidney *at operation* is a very valuable method of localizing the stone, and quite simple to carry out.

MR. W. A. JACKMAN asked what was Mr. Adams's guide as to the quantity of sodium bromide used. Pain was very variably tolerated by different patients, and if this was the guide it might produce considerable variety in normal pyelograms.

MR. DUNCAN-WOOD inquired whether Mr. Adams preferred cocaine or novocaine for urethral anæsthesia, and suggested that caudal anæsthesia might be helpful. He described a case of hæmaturia coming on at 16 years of age so severe that the kidney had to be removed, and after its removal no cause for the bleeding could be found.

DR. NOBLE asked if there was any definite pyelographic appearance characteristic of hypernephroma, and whether Mr. Adams used a manometer while at work.

---

The fifth meeting of the session was held on Wednesday, 12th February, 1929, the President in the Chair.

MR. H. S. SOUTTAR read a paper on "**The Use of Radium in Surgery,**" an abstract from which is reported on page 19 of this issue.

---

The sixth meeting of the session was held on Wednesday, 12th March, 1929, the President in the Chair.

PROFESSOR BUCKMASTER, DR. HICKMAN and MISS WILTSHIRE demonstrated experiments illustrating the physiology of the heart.

---

### Clinical Society of Bath.

A MEETING of the Clinical Society of Bath was held at the Royal United Hospital on Friday, 1st February. In the absence of the Past-President, Mr. Blathwayt, the Chair was taken by Mr. A. L. Fuller.

The following cases were shown :—

1. A case of **Raynaud's Disease of twenty-five years' duration in a woman aged 54** was shown by DR. BEVIR, of Timsbury. There was intense pain in the finger-tips, and some superficial ulceration which never entirely healed. The maximum systolic pressure was 220 mm. ; there was no albuminuria. Various treatments had been tried.

2. A specimen of **Twins Stillborn at Six Months** was shown by DR. VAISEY, of Bradford-on-Avon. X-rays showed marked disparity between the bony development of the two specimens.

3. Post-mortem demonstrations were given by MR. LEVIS on a case of **Inoperable Hypernephroma involving the Right Renal Vein,** and a case of **Stenosis of the Ureter at the Entrance to the Bladder, with resulting Dilatation of the Ureter and Hydronephrosis.**

4. A specimen of **Aortic Stenosis** was shown by DR. GORDON.

5. DR. WATERHOUSE showed a case of **Rigidity and Tremor in a girl aged 9.** The diagnosis depended very largely on the history, which was not forthcoming. There were fine tremors in both hands and general rigidity, but no pyramidal involvement. There was irregular overaction of the facial muscles. He was inclined to regard the case as either a post-encephalitic condition or as an instance of Kinnier Wilson's progressive lenticular degeneration, though the latter would be rare in so young a child. There were no signs of liver involvement, although in the case recorded of three children in one family cirrhosis of the liver preceded the lenticular degeneration. He was much struck with the facial condition.

DR. GORDON was inclined to regard it as due to lenticular degeneration; the cirrhotic symptoms might come on later. If post-encephalitic, it was not typical, because the mental deterioration had been more or less smoothly progressive rather than occurring in "steps and stairs," as was more usual in post-encephalitic states.

DR. WATERHOUSE agreed that lenticular degeneration was more probable.

6. MR. FULLER showed: (1) Two cases of **Fractured Femur,** which he had wired with aluminium bands, such as are used in fixing the control wires of a motor cycle. There was excellent resulting alignment in each case. (2) A case of **Angioma of the Forearm in a man aged 56,** in which there had been profuse bleeding. There had been an unsuccessful attempt at removal. Asked if injection treatment would be likely to be successful, MR. LEVIS preferred complete excision and skin grafting.

DR. PRESTON KING read a paper entitled "**The Unfit.**" He pointed out the serious financial burden laid on the shoulders of the fit by the ever-increasing numbers of the unfit. He included under the latter term asylum inmates, the mentally deficient, ordinary hospital patients and patients treated under the National Health Insurance Act. He considered that the money spent in training the mentally deficient would be better spent in training A1 children. He criticised the National Health Insurance Act as putting a premium on sickness, and deplored the tendency of the psycho-analyst to regard crime as the result of insanity. He advocated certification of fitness in the case of all would-be parents, a system of birth control that should be something more than mere prevention of birth, and also that syphilis should become a compulsorily notifiable disease.

---

A Meeting of the Clinical Society of Bath was held on Friday, 1st March, at the Royal United Hospital, Bath, the past President, Mr. Blathwayt, being in the Chair.

DR. SUTHERLAND showed a case of **Unexplained Chronic Headache of many years' standing occurring in a man aged 35** as a result of war injury in 1914. Wassermann negative. The question of Paget's disease was considered, but the X-ray picture showed no thickening of the bones of the skull, and in view of the history the pension factor was thought to have an important bearing on the symptoms. He also showed X-ray photographs of a case of **Myositis Ossificans.**

DR. DELICATI showed a case of **Long-standing Retention of Urine in a comparatively young man.** None of the ordinary causes for this condition could be assigned. It was suggested that there might be some disease in the neighbourhood of the filum terminale. The distribution of pain in this case might bear out this diagnosis.

DR. WATERHOUSE showed a case of **Apparent Amyotrophic Lateral Sclerosis,** in which, however, there was girdle pain and a disturbance of the temperature sense, but not of the pain sense. These otherwise inexplicable symptoms were thought to be probably functional.

MR. MUMFORD showed a specimen of a **Meningocele** recently removed from an infant with spina bifida.

MR. LEVIS showed specimens of (1) **Cystic Disease of Kidneys and Liver,** (2) **Glioma of Cerebellum in a boy aged 5,** (3) **Glioma of Cerebrum in a boy aged 9.**

DR. VINCENT COATES showed X-rays of a case of **Achalasia Gastrica,** and also read a paper dealing with the **Bio-chemical and Bio-physical Aspects of Infective Arthritis.** After reference to disorders showing Mendelian dominant and recessive characters, he proceeded to give statistics concerning the family predisposition to infective arthritis and rheumatic fever, followed by a short account of the bio-chemical tests he had found most useful and likely to yield results both from the diagnostic and therapeutic points of view.

